# Yb^2+^-Doped Silicate Glasses as Optical Sensor Materials for Cryogenic Thermometry

**DOI:** 10.3390/s24010248

**Published:** 2023-12-31

**Authors:** Hicham El Hamzaoui, Igor Razdobreev, Monika Cieslikiewicz-Bouet, Andy Cassez, Vincent Andrieux, Mohamed Bouazaoui

**Affiliations:** Univ. Lille, CERLA, CNRS, UMR 8523-PhLAM-Physique des Lasers Atomes et Molécules, F-59000 Lille, Francemonika.bouet@univ-lille.fr (M.C.-B.); andy.cassez@univ-lille.fr (A.C.); vincent.andrieux@univ-lille.fr (V.A.); mohamed.bouazaoui@univ-lille.fr (M.B.)

**Keywords:** optical sensors, divalent ytterbium, silicate glasses, cryogenic temperatures, photoluminescence, emission lifetime

## Abstract

Optical sensors constitute attractive alternatives to resistive probes for the sensing and monitoring of temperature (*T*). In this work, we investigated, in the range from 2 to 300 K, the thermal behavior of Yb^2+^ ion photoluminescence (PL) in glass hosts for cryogenic thermometry. To that end, two kinds of Yb^2+^-doped preforms, with aluminosilicate and aluminophosphosilicate core glasses, were made using the modified chemical vapor deposition (MCVD) technique. The obtained preforms were then elongated, at about 2000 °C, to canes with an Yb^2+^-doped core of about 500 µm. Under UV excitation and independently of the core composition, all samples of preforms and their corresponding canes presented a wide visible emission band attributed to Yb^2+^ ions. Furthermore, PL kinetics measurements, recorded at two emission wavelengths (502 and 582 nm) under 355 nm pulsed excitation, showed an increase, at very low *T*, followed by a decrease in lifetime until room temperature (RT). A modified two-level model was proposed to interpret such a decay time dependence versus *T*. Based on the fit of lifetime data with this model, the absolute (*S_a_*) and relative (*S_r_*) sensitivities were determined for each sample. For both the preform and its corresponding cane, the aluminophosphosilicate glass composition featured the highest performances in the cryogenic domain, with values exceeding 28.3 µsK^−1^ and 94.4% K^−1^ at 30 K for *S_a_* and *S_r_*, respectively. The aluminophosphosilicate preform also exhibited the wider *T* operating range of 10–300 K. Our results show that Yb^2+^-doped silicate glasses are promising sensing materials for optical thermometry applications in the cryogenic domain.

## 1. Introduction

Due to the high optical conversion efficiency of trivalent ytterbium ions (Yb^3+^) in the near-infrared (NIR) domain, Yb^3+^-activated silicate glasses are among the most interesting luminescent materials [1]. Moreover, compared to their crystalline counterpart, these glassy materials show higher mechanical and thermal stabilities, enabling their drawing and shaping as optical fibers. This made the development of high-power fiber lasers possible for several applications, including materials processing, medicine and telecommunications [2,3]. In addition, in silicate matrices, ytterbium ions can also exist in a divalent state (Yb^2+^). The relative concentration of both kind of ions within the host material largely depends on the oxidizing/reducing conditions during the material fabrication [4]. However, although Yb^2+^ ions present an interesting visible photoluminescence (PL) under UV excitation, their presence could be critical for laser applications [5,6]. This could explain why this visible optical activity of Yb^2+^ ions has hardly been studied in glasses and was limited, at RT, to white light source applications [7]. In the field of temperature sensing, and for applications where conventional thermometry is difficult to implement, it is required to monitor the temperature in volume-restricted areas. This has prompted the development of remote temperature measurement methods based on optical fibers [8]. The former offer key advantages compared to conventional sensors: small size, flexibility, intrinsic immunity to electromagnetic interference and their ability for remote interrogation. Among the luminescence-based techniques, lifetime thermometry is commonly used, as it is not sensitive to external factors, such as excitation fluctuations, obviating the need for intensity referencing and, thus, minimizing the associated errors [9]. Based on this approach, Yb^3+^-activated optical fibers have demonstrated their potential as probing materials for thermometric applications from RT to 973 K [10]. Nevertheless, Yb^3+^ ions are less suitable for lower temperature sensing due to the small change in their lifetime with temperature, particularly in the cryogenic domain [10,11]. On the other hand, for thermometry in this domain, until now, investigations have concerned mainly Yb^2+^ ions in fluorite-type crystals such as CaF_2_ and SrF_2_. Indeed, the fluorescence decay time of such Yb^2+^-doped materials is temperature-sensitive above all in the cryogenic range [12]. The association of such crystalline materials with optical fibers has led to cryogenic temperature punctual sensors in the 20–120 K range [13]. Hitherto, to the best of our knowledge, the thermal behavior of Yb^2+^ ion fluorescence in silicate glass matrices has not been investigated for cryogenic thermometry. Furthermore, from the perspective of further reducing the sensing material size and thus increasing its spatial resolution without interfering with the measured object itself, an alternative to the use of a crystalline material at the end of an optical fiber could be the use of a more compatible active glassy one. Therefore, the size of the sensing material could be easily adjusted by drawing and then connecting to a transport optical fiber by conventional glass fusion splicing. This also offers, in the case of small, measured objects, the possibility to use the sensor while keeping the temperature in equilibrium during the measurement. In order to reach this advanced stage in development, upstream investigations are needed. Hence, in this paper, Yb^2+^-doped silicate optical preforms were prepared using the MCVD method in reducing conditions. The obtained preforms were also elongated, at about 2000 °C, to canes with Yb^2+^-doped cores of about 500 µm. The thermal behavior of Yb^2+^ ion luminescence, under UV excitation, has been evaluated in the temperature range of 2–300 K.

## 2. Materials and Methods

Yb-doped aluminophosphosilicate (YAPS) and aluminosilicate (YAS) optical preforms were fabricated, using the MCVD process in combination with the solution doping (SD) technique, at the FiberTech Lille platform (PhLAM laboratory, Lille, France). Initially, a pure silica tube (F300, Heraeus, Hanau, Germany) was mounted on MCVD lathe. Then, this tube was etched by inside flowing of C_2_F_6_, O_2_ and He gases to clean up its inner wall at 1900 °C. Subsequently, one sintered barrier layer of pure silica was deposited there, using SiCl_4_ with O_2_ at 2000 °C, followed by the deposition of one non-sintered silica soot layer at 1690 °C applying a gaseous flow of SiCl_4_ with O_2_ and He. After cooling, the tube was maintained under a controlled atmosphere and placed for vertical SD using a solution of YbCl_3_ in ethanol, along with AlCl_3_·6H_2_O for the YAS composition. In the case of the YAPS ones, we used a solution of diluted phosphoric acid, along with AlCl_3_·6H_2_O and YbCl_3_. The soot layers were soaked for 40 min, and after the impregnation, the solution was drained out and the tube was fixed vertically and kept in a controlled neutral atmosphere for drying. Afterward, the tube was mounted on an MCVD lathe and dried again by a flow of a hot mixture of Cl_2_ and He gases around 1000 °C. Then, a stepwise sintering to sealing was done in full H_2_/Ar atmosphere with an increasing temperature from 1700 °C to 2150 °C.

The Yb-doped glassy preforms were drawn at a temperature of about 2000 °C down to a millimeter-sized cane with doped core of about 500 µm. The fabricated preforms and their corresponding drawn canes were cut and polished for optical characterizations.

UV–Visible absorption spectra were obtained at room temperature using a Cary 5000 double-beam spectrophotometer (Agilent, Santa Clara, CA, USA).

PL measurements in the temperature range of 2–300 K were achieved in the closed cycle magneto optical cryostat SpectromagPT (Oxford Instruments, Oxford, UK). The time-resolved PL spectra and PL decay kinetics were recorded under 355 nm pulsed excitation in the photon-counting regime with the use of a single-grating monochromator M266 (Solar LS) equipped with a thermoelectrically cooled GaAs(Cs) photomultiplier R943-02 (Hamamatsu Inc., Hamamatsu City, Japan) and multistop scaler P7887 (FAST ComTec GmbH, Oberhaching, Germany) with a time resolution up to 250 ps. The pulsed source of excitation at 355 nm was the pump laser integrated into the optical parametric oscillator (OPO) Opolette 355 LD (Opotek Inc., Carlsbad, CA, USA), with a tunable repetition rate (up to 100 Hz) and 8 ns pulse duration.

## 3. Results and Discussion

**Figure 1:**
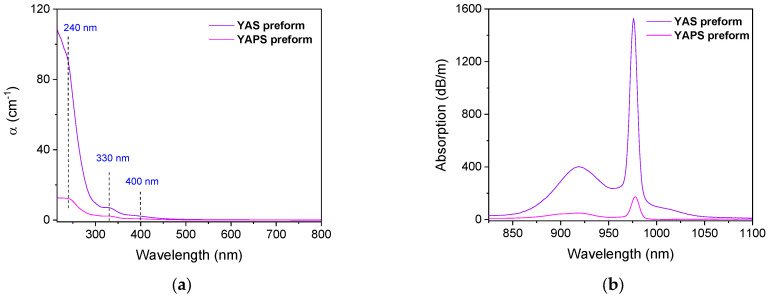
(**a**) UV–Visible and (**b**) NIR optical absorption spectra of YAPS and YAS preforms.

Figure 1 shows the optical absorption spectra of the YAPS and YAS preforms. They present, in the UV–Visible range (Figure 1a), three bands peaking at 240, 330 and 400 nm. The bands around 330 nm and 400 nm are due to divalent Yb^2+^ ions and originate from the 4f^14^→4f^13^5d transitions. These attributions are based on comparisons with similar absorption bands reported in the case of Yb/Al/P- and Yb/Al-doped silica glasses [4,14]. The last one centered at 240 nm can be assigned to charge transfer transitions between the Yb^3+^ ion and the oxygen ligands in the glass network [5,14]. In addition, the absorption bands shown in Figure 1b, in the NIR wavelength range of 825–1100 nm, are assigned to the ^2^F_7∕2_ → ^2^F_5∕2_ transitions of Yb^3+^ ions [4]. Therefore, both Yb^2+^ and Yb^3+^ ions coexisted in our YAPS and YAS samples.

From the value of absorption at the wavelength of 977 nm, the concentration of Yb^3+^ ions could be estimated using the absorption cross-section reported in the literature for Yb-doped aluminophosphosilicate and aluminosilicate glasses [15]. Moreover, based on the mean Yb concentration determined from the electron probe microanalysis (EPMA), the concentration of Yb^2+^ ions was also evaluated. The obtained ytterbium ion concentrations with those of the Al and P elements, determined from EMPA, are compiled in Table 1. We would like to stress that the choice here of aluminophosphosilicate and aluminosilicate glasses was related to their belonging to two families of silicate glasses, in which their compositions are commonly used in active optical fiber fabrication. Moreover, an optimization of these glass compositions for thermometry applications is outside the scope of this work.

**Figure 2:**
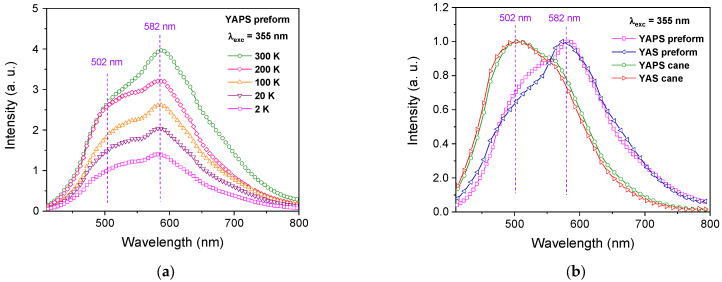
PL spectra, under 355 nm pulsed excitation, of (**a**) YAPS preform measured at different temperatures and (**b**) YAPS and YAS preforms with those of their corresponding canes recorded at 2 K. The PL spectra were obtained by the time integration of the time-resolved ones.

Figure 2a shows the luminescence spectra of the YAPS preform, recorded at different temperatures under excitation at 355 nm. Figure 2b displays the PL spectra of YAPS and YAS preforms, with those of their corresponding drawn canes, obtained at 2 K. All these spectra are dominated by a wide visible emission band covering almost the whole visible range. According to the current understanding of the luminescence mechanism of Yb^2+^ ions, this band is attributed to the 4f5d→4f transitions [4,5]. One can note that, independently on the temperature, the obtained PL band presents a maximum around 582 nm or 502 nm (Figure 2a). Moreover, if, for a preform sample, the PL band presents a maximum around 582 nm and a shoulder around 502 nm, the opposite is true for its corresponding drawn cane (Figure 2b). This result could be attributed to the presence of two main distinct local environments of Yb^2+^ ions inside the host glass matrix at the origin of the PL around 502 nm and 582 nm. Furthermore, while the overall PL spectral profile does not depend on the composition of the sample, the relative contribution of Yb^2+^ ions, inside each local environment, to the visible PL spectra is reversed in the drawn cane with respect to its corresponding preform. Either way, regardless of the sample and its composition, the recorded PL spectra remain structured around the two main contributions at about 502 nm and 582 nm. For this reason, thereafter, the PL kinetics were studied at these two emission detection wavelengths (λ_det_) under excitation at 355 nm (λ_exc_).

**Figure 3:**
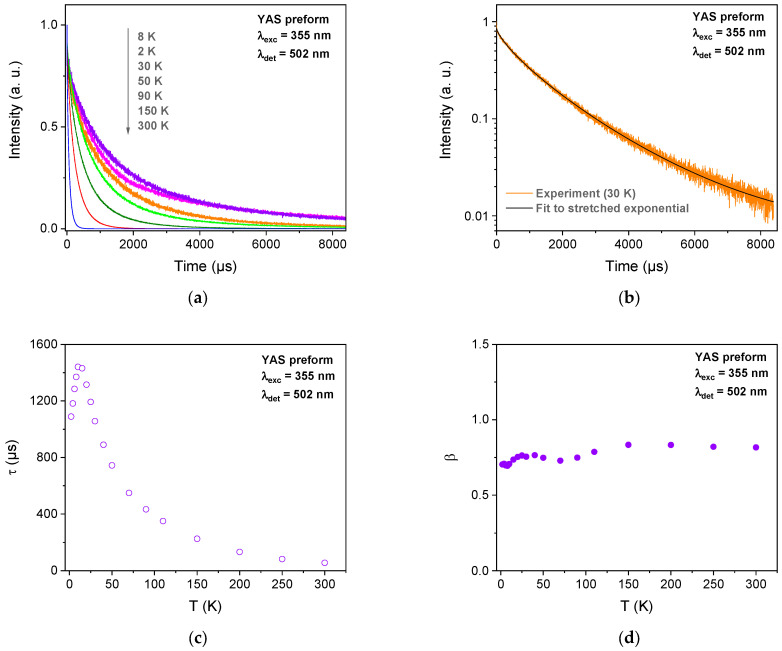
(**a**) PL kinetics recorded at 502 nm, under excitation at 355 nm, of YAS preform for different temperatures. (**b**) PL decay curve at 30 K and its corresponding curve fitting analysis using Equation (1). The obtained *τ* (**c**) and *β* (**d**) values are a function of the temperature.

In Figure 3a, we presented the results of the PL decay measurements performed on YAS preform at different temperatures in the range from 2 to 300 K. PL kinetics were recorded at 502 nm under excitation at 355 nm. Independently, on the T, all the obtained PL decay curves do not follow a pure mono-exponential function. Nevertheless, they could be successfully fitted by a stretched exponential function that reflects the multisite environment of the Yb^2+^ ions [16,17]:

(1) $$I(t)=y_{0}+A\exp\left[-{\left(\frac{t}{\tau }\right)}^{\beta }\right]$$

where *I*(*t*) is the luminescence intensity, *t* is time, *y*_0_ is the background (noise level), *A* is the intensity at *t* = 0, *β* is the stretch factor and *τ* is a characteristic relaxation time. The decay curve and a fit to the function of Equation (1) are shown as an example for *T* = 30 K in Figure 3b. The common interpretation of such a stretched exponential behavior refers to the overall relaxation of a glass containing a number of independently relaxing species. Every species decays exponentially with one specific relaxation rate *τ* [18]. It is known that the presence of a co-dopant such as aluminum or phosphorus enhances the dispersion of rare-earth ions inside the glass matrix [19,20]. It reduces their ability for cluster formation and leads to disordered local environments with a multiplicity of sites available for the rare-earth ions. Hence, as our sample contains Al/P or Al, this could explain the stretched exponential nature of the PL dynamic observed at 502 nm. The obtained *τ* values, as function of *T*, are presented in Figure 3c and the corresponding *β* ones in Figure 3d. If this former parameter remains within the range of 0.67–0.79, *τ* values start with an increase at very low *T* before a decrease until RT.

**Figure 4:**
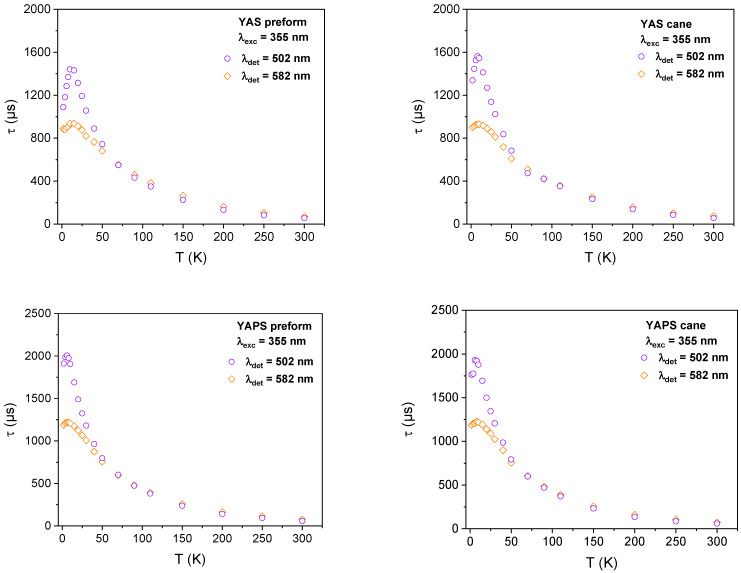
*τ* values as function of the temperature for YAS and YAPS preforms and their corresponding elongated canes, obtained at 502 and 582 nm emission wavelengths under 355 nm.

In Figure 4, we presented *τ* values evolution as a function of the temperature for YAS and YAPS preforms and their corresponding canes obtained at two emission wavelengths (502 and 582 nm) under 355 nm excitation. The trend of this PL lifetime is wavelength-dependent. Indeed, at a 502 nm emission wavelength, the emission lifetime is highly temperature-sensitive in the range of 10–50 K when compared to 582 nm. Moreover, this sensitivity is higher with the YAPS samples compared to the YAS ones. One can also note that, for each kind of glass (YAS or YAPS), similar temperature-dependent features manifest in the preform and its corresponding elongated cane. This behavior attests the preservation of emitting Yb^2+^ ions in the cane samples after drawing at about 2000 °C.

In addition, we tried to model the observed temperature dependence of luminescence lifetimes. We note here that the two-level model, used in the case of Yb^2+^-activated crystals [12], could not predict the first increase in the fluorescence lifetime occurring at a very low *T* below 10 K.

**Figure 5:**
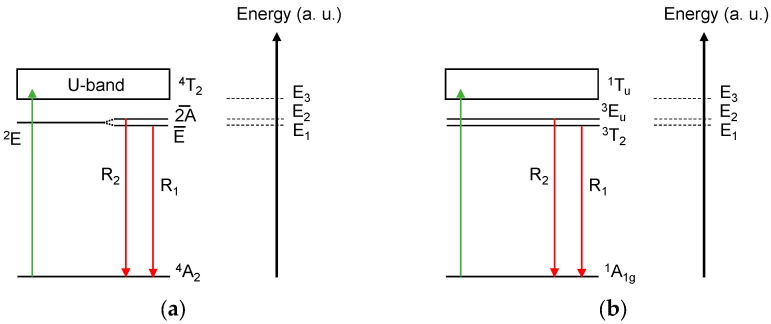
Simplified energy level diagrams of (**a**) Cr^3+^ ions in oxide crystals and (**b**) Yb^2+^ ions in octahedral symmetry (O_h_) sites adapted from [22,24], respectively. Colored vertical arrows show excitation (in green) and emission (in red) transitions.

On another note, our samples show a global trend of *τ* as function of *T* similar to the ones observed, firstly, with Cr^3+^ ions in oxide crystals [21,22] and, more recently, with Mn^4+^ ions [23]. Indeed, the decay time constant exhibits an initial increase with cooling. Then, it reaches a maximum value, followed by a decrease with further cooling. Based on the energy level diagram of Cr^3+^ ions in oxide crystals (Figure 5a), a modified two-level model has been also proposed, which enabled an understanding of their fluorescence lifetime dependences versus *T* [21,22]. The evolution of the fluorescence lifetime versus temperature, with the maximum at low temperatures, was explained assuming different radiative transition probabilities from the split levels $\bar{E}$ and $\bar{2A}$ (Figure 5a). The probability of radiative decay from the upper level $\bar{2A}$ is lower compared to that of $\bar{E}$. This induces an increase in the measured radiative decay time of the R-lines emission. Nevertheless, a decrease in the temperature leads to depopulation of the upper level $\bar{2A}$. This results in the reduction of its contribution to the emission process in favor of transitions from the low-lying level $\bar{E}$, with a higher radiative decay rate. Thereafter, this provokes a decrease in the measured R-lines emission decay time with further cooling. The modified two-level model includes the main processes affecting the population and radiative rates of the ^2^E state, namely thermalization, phonon-assisted interactions with lattice vibrations and thermally induced depopulation. According to this model [22], the fluorescence lifetime *τ* is given by Equation (2):

(2) $$\tau \;(T)=\frac{\sum_{i=1}^{3}g_{i}\exp\left(\frac{{\Delta E}_{i}}{kT}\right)}{\sum_{i=1}^{3}g_{i}R_{i}\exp\left(\frac{{\Delta E}_{i}}{kT}\right)}$$

where *R_i_* are the radiative decay rates, *g_i_* are degeneracies of the states, Δ*E_i_* is the energy difference between the *i*th state and the lower excited level, *k* is the Boltzmann constant and *T* is the absolute temperature.

By considering the phonon-induced interactions, the radiative decay rate of the *i*th state was expressed by Equation (3):

(3) $$R_{i}=\frac{1}{\tau_{i}}coth\left(\frac{E_{p}}{2kT}\right)$$

where *τ_i_* is the radiative decay time constant, and *E_p_* stands for “effective energy” of the phonons responsible for the exchange with the sidebands [22].

In addition, both Yb^2+^ and Cr^3+^ ions have comparable energy level structures (Figure 5b). Indeed, in an O_h_-symmetric environment, the Yb^2+^ ions have ^1^A_1g_, ^3^T_2_/^3^E_u_ and ^1^T_1u_ as the ground state, the first excited split levels and the second excited level, respectively [24]. This similarity has prompted the consideration of Equation (2) in fitting the results obtained with our Yb^2+^-doped samples. In the case of Yb^2+^, the integers *i =* 1, 2 and 3 correspond to the ^3^T_2_, ^3^E_u_ and ^1^T_1u_ levels with degeneracy equal to 9, 6 and 3, respectively. Subsequently, Δ*E*_1_ = 0, Δ*E*_2_ = *D*, the energy split of the ^3^T_2_/^3^E_u_ levels and Δ*E*_3_ = Δ*E* is the gap between the ^3^E_u_ and ^1^T_1u_ levels. We also introduced two simplifications, as for Cr^3+^ in [22]. In the first one, we assumed the use of the same value of the phonon energy *E_p_* for the ^3^T_2_ and ^3^E_u_ levels due to their proximity. In the second one, we omitted the phonon-induced interaction for the radiative decay transitions occurring from the level ^1^T_1u_. Thus, the expression for *τ* (*T*) can be rearranged as follows:

(4) $$\tau \;\left(T\right)=\frac{1+\frac{2}{3}\exp\left(-\frac{D}{kT}\right)+\frac{1}{3}\exp\left(-\frac{\Delta E}{kT}\right)}{\frac{1}{\tau_{1}}coth\left(\frac{E_{p}}{2kT}\right)+\frac{2}{{3\tau }_{2}}coth\left(\frac{E_{p}}{2kT}\right)exp\left(-\frac{D}{kT}\right)+\frac{1}{{3\tau }_{3}}\exp\left(-\frac{\Delta E}{kT}\right)}$$

In this case, *τ*_1_, *τ*_2_ and *τ*_3_ are the radiative decay times of the ^3^T_2_, ^3^E_u_ and ^1^T_1u_ levels, respectively.

**Figure 6:**
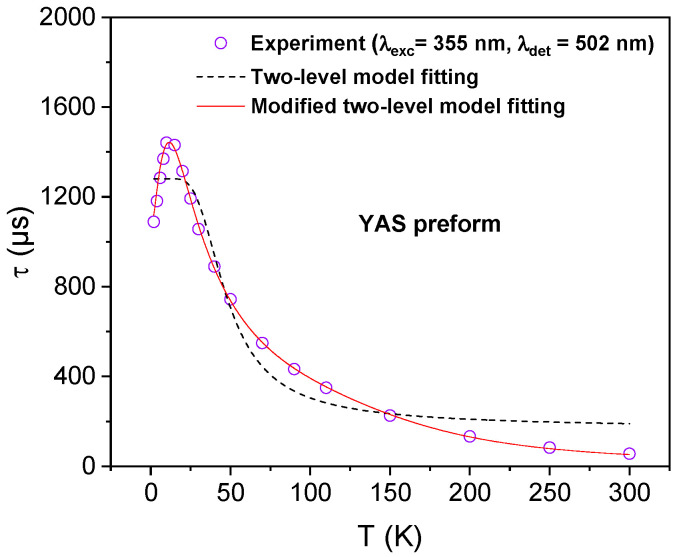
Fitting curves to the experimental results of *τ* as function of T in the case of the YAS preform using the equation related to the two-level model reported in [12,21] and Equation (4) associated with the modified two-level model.

Equation (4) was then used to fit the experimental data. As shown for the example of YAS sample in Figure 6, Equation (4) (red curve) fits well the experimental temperature dependence of the decay time. It indicates that the modified two-level model successfully explains the behavior of the Yb^2+^ ion fluorescence lifetime evolution over the entire investigated temperature range. Moreover, the use of the equation, deduced from the two-level model reported in [12,21] and expressing the fluorescence lifetime as a function of temperature, did not allow a good fit of the experimental data in the decreasing zone after 10 K (the dashed black curve in Figure 6).

The obtained fit parameters, using the modified two-level model, of the Yb^2+^-doped samples are summarized in Table 2.

From Table 2, we note that, regardless of the sample nature (preform or cane) and λ_det_, the *D* value is very low compared to the Δ*E* one. This shows that the energy gaps between the split levels of Yb^2+^ ions (^3^T_2_ and ^3^E_u_) are weak compared to the one separating the first and third excited states (^3^T_2_ and ^1^T_1u_). Furthermore, for all the samples, the obtained *E_p_* phonon energy was in the 19–40 cm^−1^ range. This indicates the involvement of low-energy phonons in Yb^2+^ ion–phonon coupling in our samples. These *E_p_* values are located in the low-frequency region dominated by the boson peak (BP), a feature characteristic of the glass medium-range order [25,26]. The BP was interpreted as reflecting the acoustic vibration of cohesive domains [27]. Such vibrations could be involved in the phonon coupling with Yb^2+^ ions in our samples. Concerning the radiative decay times, we obtained *τ*_3_ < *τ*_1_ < *τ*_2_ for all the samples. *τ*_1_ < *τ*_2_ agrees with the reasoning underlying the interpretation of the observed change in the luminescence decay time constant at low temperatures. On the other hand, *τ*_3_ < *τ*_1_ and *τ*_2_ is consistent with the evolution of the fluorescence lifetime at high temperatures. We would like to stress here that a refined understanding of the obtained parameter values in association with the sample nature (preform or cane), glass composition or λ_det_ deserves further investigation, which is outside the scope of the present study.

From a practical standpoint, our Yb^2+^-doped samples exhibit a lifetime evolution versus *T*, with a maximum around 10 K. In contrast, for other materials such as Cr-doped crystals [22], this maximum was found at higher temperatures. In our case, the temperature dependency is more suitable for thermometric applications. Indeed, a temperature dependence, with the maximum at higher temperatures, will cause an indistinctness of the temperature reading and thus limits the operating cryogenic range of the sensor. From the experimental results (Figure 2), we estimated the operating cryogenic ranges of our samples. In these ranges for each temperature corresponds one luminescence lifetime *τ* value. As depicted in Table 3, independently of the emission detection wavelength, the YAPS samples present larger temperature operating ranges when compared to the ones of YAS composition. For further comparison, related to the performances of our samples for thermometry, we calculated the absolute and relative sensitivity, *S_a_* and *S_r_*, using Equations (5) and (6), respectively. These parameters are defined as follows [28]:

(5) $$S_{a}=\left|\frac{𝜕\tau }{𝜕T}\right|$$

(6) $$S_{r}=\left|\frac{1}{\tau }\frac{𝜕\tau }{𝜕T}\right|\times 100\%$$

In Table 3 are also summarized the obtained values of *S_a_* and *S_r_* at three different temperatures.

As can be seen from Table 3, both *S_a_* and *S_r_* values decrease with the temperature for all the samples. We also note that, for a given glass composition, YAS or YAPS, the calculated parameters are rather more dependent on the emission wavelength than on the drawing of the preform on a cane. Moreover, regardless of the temperature, the sample and its composition, the *S_a_* and *S_r_* values obtained at a 502 nm emission wavelength are higher compared to the ones at 582 nm. At 502 nm, the composition that stands out is YAPS. It shows a larger temperature operating range of 10–300 K and 15–300 K for the corresponding preform and cane, respectively. Furthermore, both of them also present the highest sensitivities, exceeding the values of 28.3 µsK^−1^ and 94.4% K^−1^ at 30 K for *S_a_* and *S_r_*, respectively. Our samples exhibit a comparable sensitivity to Yb^2+^ ions in fluorite-type crystals [13] and a higher one compared to Cr^3+^-doped Ga_2_O_3_ or Mn^4+^-activated Li_2_TiO_3_ [22,28]. With respect to these three phosphors, they present a wider T-sensing operating range. We also note that the thermometry performances of each preform and its corresponding cane were maintained after drawing at 2000 °C, particularly in the case of a YAPS composition with a maximum relative standard deviation of about 1% for both *S_a_* and *S_r_* at 30 K.

## 4. Conclusions

We fabricated two kinds of Yb^2+^-doped preforms, with aluminosilicate and aluminophosphosilicate core glasses, using the MCVD technique under reducing conditions. These preforms were then elongated, at about 2000 °C, to canes with an Yb^2+^-doped core of about 500 µm.

Under UV excitation, all samples presented a wide visible emission band attributed to Yb^2+^ ions. The PL kinetics measurements, recorded at two emission wavelengths (502 and 582 nm) under 355 nm pulsed excitation, showed an increase at very low *T*, followed by a decrease in the lifetime until room *T*. We extended the modified two-level model to explain the fluorescence lifetime evolution as a function of the temperature. The validity of this model was demonstrated through its close fit to the obtained lifetime data, which also gave access to the expressions of *τ* as a function of *T* for each Yb^2+^-doped sample. Based on these expressions, we calculated the absolute and relative sensitivities of each sample. They were dependent on the emission wavelength, with higher performances at 502 nm. The aluminophosphosilicate glass composition presented the highest sensitivities in the cryogenic domain and the wider *T* operating range. Furthermore, after drawing, the optical activity of the Yb^2+^ ions in the visible range and thermometric performances were preserved in the cane samples. Moreover, compared to Yb^2+^ ions in fluorite-type crystals, our samples present a wider T-sensing operating range.

In addition, both Yb^2+^ and Yb^3+^ ions coexist in our YAPS and YAS samples. Although it remains to be determined whether the presence of Yb^3+^ ions has an impact on the thermal behavior of the Yb^2+^ luminescence decay time in these samples, from a practical point of view, the presence of trivalent ions (Yb^3+^) would be an asset that can be exploited for high-temperature thermometry. Hence, this will pave the way for efficient *T* sensing with both Yb^2+^/Yb^3+^-activated glasses in wider *T* operating ranges, starting from cryogenic temperatures to those beyond 300 K.

## Figures and Tables

**Table 1:** Estimated mean concentrations of Yb^2+^/Yb^3+^ ions and those of the Al and P elements, determined using EPMA, inside the fabricated preforms.

Sample	(Yb^2+^) (cm^−3^)	(Yb^3+^) (cm^−3^)	(Al) (at.%)	(P) (at.%)
YAS preform	8 × 10^19^	1.4 × 10^20^	1.78	-
YAPS preform	1 × 10^19^	1.9 × 10^19^	0.62	0.23

**Table 2:** Fit parameters obtained from the temperature dependence of the luminescence decay time using Equation (4) for all samples.

Sample	λ_det_ (nm)	*τ*_1_ (µs)	*D* (cm^−1^)	Δ*E* (cm^−1^)	*E_p_* (cm^−1^)	*τ*_2_ (ms)	*τ*_3_ (µs)
YAS preform	502	1090	4.5	616	32.0	95	0.9
YAS cane	502	1340	6.6	764	23.6	67	0.5
YAS preform	582	877	20.2	677	44.1	67	1.1
YAS cane	582	909	21.5	712	39.3	83	1.1
YAPS preform	502	1750	10.4	574	19.4	67	1.5
YAPS cane	502	1920	8.8	560	21.5	95	1.3
YAPS preform	582	1180	20.9	541	34.9	67	2.4
YAPS cane	582	1180	19.5	530	35.1	83	2.2

**Table 3:** Temperature operating ranges and calculated *S_a_* and *S_r_* values at different *T* using Equation (6) for all samples.

Sample	λ_det_ (nm)	*T* Operating Range	*S_a_* (µs K^−1^) @30 K	*S_a_* (µsK^−1^) @70 K	*S_a_* (µsK^−1^) @300 K	*S_r_* (%K^−1^) @30 K	*S_r_* (%K^−1^) @70 K	*S*r (%K^−1^) @300 K
YAS preform	502	30–300 K	22.22	7.12	0.38	74.09	10.17	0.12
YAS cane	502	20–300 K	24.00	6.49	0.45	80.00	9.28	0.15
YAS preform	582	25–300 K	9.32	5.73	0.50	31.06	8.19	0.16
YAS cane	582	20–300 K	10.64	5.39	0.49	35.48	7.71	0.16
YAPS preform	502	10–300 K	28.32	7.45	0.41	94.42	10.65	0.13
YAPS cane	502	15–300 K	28.37	7.67	0.38	94.59	10.96	0.12
YAPS preform	582	15–300 K	15.35	6.64	0.46	51.16	9.48	0.15
YAPS cane	582	20–300 K	15.58	6.79	0.43	51.94	9.71	0.14

## Data Availability

The data presented in this study are available on request from the corresponding author. The data are not publicly available due to privacy restrictions.
